# Antibodies induced by enterotoxigenic *Escherichia coli* (ETEC) adhesin major structural subunit and minor tip adhesin subunit equivalently inhibit bacteria adherence *in vitro*

**DOI:** 10.1371/journal.pone.0216076

**Published:** 2019-05-01

**Authors:** Hyesuk Seo, Rahul M. Nandre, Jerome Nietfeld, Zhenhai Chen, Qiangde Duan, Weiping Zhang

**Affiliations:** 1 Department of Diagnostic Medicine/Pathobiology, Kansas State University College of Veterinary Medicine, Manhattan, Kansas, United States of America; 2 University of Illinois at Urbana-Champaign, Department of Pathobiology, Urbana, Illinois, United States of America; New York State Department of Health, UNITED STATES

## Abstract

Antibodies that block the adherence of enterotoxigenic *Escherichia coli* (ETEC) to host intestinal epithelial cells are protective. Multiepitope-fusion-antigens (MEFAs) carrying epitopes of ETEC adhesin major subunits or tip minor subunits induced antibodies against ETEC adherence. Adherence inhibition effectiveness of antibodies induced by major subunit epitopes versus minor tip subunit epitopes, however, has not been comparatively characterized. In this study, we immunized mice with a major subunit MEFA or a tip MEFA, evaluated MEFA anti-adhesin immunogenicity, and examined induced-antibodies against bacteria *in vitro* adherence or *in vivo* colonization in mice. Mice subcutaneously immunized with major subunit MEFA CFA/I/II/IV or tip MEFA showed no adverse effects and developed strong antigen-specific antibody responses. Data showed that antibodies derived from two MEFAs were equally effective against adherence of the bacteria expressing CS1, CS2, CS3, CS4/CS6, CS5/CS6, or CS6 adhesin *in vitro*. Subsequently, we immunized mice with CFA/I fimbriae, major subunit CfaB, or minor tip adhesin subunit CfaE. We found that antibodies induced by CFA/I, CfaB and CfaE equally inhibited *in vitro* adherence of ETEC strain H10407. Furthermore, we immunized mice with CFA/I fimbriae, CfaB, or CfaE, and then challenged the mice with H10407. Data showed that although not significantly, fewer H10407 bacteria colonized the immunized mice. These results suggest that ETEC adhesin major subunit and minor tip subunit should be equally effective in inducing neutralizing anti-adhesin antibodies, and that major subunit CFA/I/II/IV MEFA or tip MEFA, perhaps combined with toxoid fusion 3xSTa_N12S_-mnLT_R192G/L211A_, can be used for development of broadly protective vaccines against ETEC diarrhea.

## Introduction

Diarrheal disease remains a major global health problem as it continues to be a leading cause of death in children younger than 5 years of age in the developing countries [1, 2]. Enterotoxigenic *Escherichia coli* (ETEC) bacteria are among the leading causes of children’s diarrhea [1–3]. ETEC associated diarrhea contributes to a significant portion of the children annual death rate [2, 4]. ETEC bacteria are also the most common cause of diarrhea in children and adults travelling from developed countries to ETEC endemic regions, civil and military personnel deployed at developing countries, and perhaps immunocompromised patients [5–7]. Unfortunately, there is no licensed vaccine against ETEC diarrhea [8, 9].

Blocking ETEC bacterial adherence to host receptors is regarded an effective prevention strategy, since adhesin-mediated bacterial attachment to host small intestinal epithelial cells is the first step of ETEC infection [10]. Anti-adhesin vaccines that prevent ETEC bacteria from adhering to host receptors and colonizing small intestines can thus serve as the first line of defense against ETEC diarrhea [8, 9, 11]. ETEC adhesins include fimbrial apparatus and non-fimbrial outer membrane proteins. A typical ETEC fimbria is composed of a major structural subunit and a few minor subunits including tip adhesin subunit, usually with hundreds or thousands of copies of a major subunit but one to a few copies of each minor subunit [12]. Fimbrial major structural subunits are the structural block of ETEC bacterial fimbriae, whereas tip adhesin subunit located at the very end of a fimbria attaches to host cell receptors. Antibodies to ETEC fimbrial major subunits or tip adhesin subunits are shown effective against ETEC bacterial adherence [8, 11, 13, 14].

We recently developed a structure- and epitope-based vaccine technology called MEFA, multiepitope fusion antigen. By applying this MEFA technology, we constructed multivalent MEFA immunogens by embeding epitopes from multiple fimbrial major subunits or minor tip adhesin subunits in a backbone ETEC subunit protein (via epitope substitution); we subsequently explored the potential of MEFAs in developing broadly protective anti-adhesin subunit vaccines against ETEC diarrhea. A major subunit MEFA named CFA/I/II/IV MEFA, which carried epitopes from the major structural subunits of seven most important ETEC adhesins (CFA/I, CS1-CS6), induced neutralizing antibodies against the adherence of all seven adhesins [15, 16]. Similarly, a tip adhesin MEFA named tip MEFA was constructed by integrating epitopes from minor tip adhesin subunits or adhesive subunits of these seven ETEC adhesins (CFA/I, CS1-CS6) as well as adhesins CS21 and EtpA. This tip MEFA also induced neutralizing antibodies against the adherence of these seven adhesins (CFA/I, CS1-CS6) plus CS21 and EtpA adhesins [17].

Unlike minor tip adhesin subunits or adhesive subunits, fimbrial major structural subunits typically do not play a direct role in attaching bacteria to host cell specific receptors, unless they are also the adhesive subunit. However, recent studies revealed that antibodies induced by major structural subunit epitopes (CFA/I/II/IV MEFA) inhibited ETEC bacterial adherence, the same as antibodies derived from minor tip adhesin epitopes (tip MEFA) [15, 17]. Mechanism of which major structural subunit-induced antibodies prevent ETEC bacteria from adherence to host receptors was not fully characterized. Additionally, efficacy against bacterial adherence from antibodies induced by major subunit CFA/I/II/IV MEFA and tip MEFA has never been comparatively examined. Moreover, it has never been examined whether antibodies to a fimbrial subunit (major or minor)and antibodies to an entire fimbria can equally block ETEC bacterial adherence. In this study, we subcutaneously immunized mice with an equivalent dose of CFA/I/II/IV MEFA, which carries epitopes of the major subunits of seven ETEC adhesins CFA/I, CS1-CS6, or tip MEFA which carries epitopes of the minor tip or adhesive subunits of the nine adhesins (CFA/I, CS1-CS6, CS21 and EtpA), and comparatively evaluated immunogenicity of two MEFA antigens. (Here we use adhesins instead of fimbriae since CS6 is an outer membrane adhesin, not a typical fimbria). We also immunized mice with entire CFA/I fimbria, CFA/I fimbrial major structural subunit CfaB, or fimbrial tip adhesin subunit CfaE to assess induced antibodies for efficacy against ETEC bacterial adherence. Additionally, we immunized mice and then challenged the immunized mice with ETEC strain H10407 to evaluate antibody protection against ETEC colonization. We further explored the potential application of CFA/I/II/IV MEFA or tip MEFA, co-administered with toxoid fusion 3xSTa_N12S_-mnLT_R192G/L211A_ (previously labeled as 3xSTa_N12S_-dmLT) which has been demonstrated to induce antibodies protecting against ETEC heat-labile toxin (LT) and heat-stable toxin (STa) [18], for vaccine development against ETEC diarrhea.

## Methods and materials

### Bacterial strains

The *E*. *coli* strains used in this study are listed in Table 1. *E*. *coli* recombinant strains expressing the major subunit MEFA (CFA/I/II/IV MEFA; 9472) which was modified by removing the 6xHis-tag from 9175 [15], tip MEFA (9450) [17], and monomeric toxoid fusion 3xSTa_N12S_-mnLT_R192G/L211A_ (9471; previously named 3xSTa_N12S_-dmLT) which was derived by deleting the 6xHis-tag of 9331 [18], were used for protein expression and extraction [17, 18]. ETEC field isolates provided by Johns Hopkins University, Washington University at St. Louis and University of Gothenburg *E*. *coli* Reference Strain Center (Sweden), and K-12 recombinant *E*. *coli* strains expressing CS1 and CS2 adhesins from Emory University were used for the extraction of bacterial fimbria (as ELISA coating antigens to titrate antibodies induced by fimbriae, a major subunit, or CFA/I/II/IV MEFA) or *in vitro* antibody adherence inhibition assays. Recombinant *E*. *coli* strains expressing tip adhesins or an individual adhesive subunit were used for extraction of proteins to be used as the coating antigens in ELISAs to titrate antibodies derived from tip MEFA or a tip subunit recombinant protein (Table 1).

**Table 1:** A list of *Escherichia coli* strains used in this study. Fimbrial *E*. *coli* and ETEC strains were used for fimbria extraction and *in vitro* antibody adherence inhibition assay. Recombinant *E*. *coli* strains expressing each tip subunit were used to express proteins as the coating antigen of ELISAs to titrate anti-tip antibodies.

Strain	Relevant characteristics	Source
H10407	O78:H11; CFA/I, LT, STa	Johns Hopkins University
THK38/pEU405	CS1	Emory University
DH5α/pEU588	CS2	Emory University
E116 (E19446)	CS3, LT, STa	University of Gothenburg
E106 (E11881/9)	CS4/CS6, LT, STa	University of Gothenburg
UM 75688	CS5/CS6, LT, STa	Johns Hopkins University
JF2423 ETP98066	CS6, LT, STa	Washington University
JF2101	CS21, EtpA, STa	Washington University
JF2318 ETP050008	EtpA, STa	Washington University
9473	CfaE (CFA/I) in pET28α/ BL21 Kan^+^	[17]
9474	CooD (CS1) in pET28α/ BL21 Kan^+^	[17]
9475	CotD (CS2) in pET28α/BL21 Kan^+^	[17]
9505	CstH (CS3) in pET28α/BL21 Kan^+^	[17]
9504	CsaE (CS4) in pET28α/BL21 Kan^+^	[17]
9533	CsfD (CS5) in pET28α/BL21 Kan^+^	[17]
9506	CssB (CS6) in pET28α/BL21 Kan^+^	[17]
9468	LngA (CS21) in pET28α/BL21 Kan^+^	[17]
9507	EtpA in pET28α/BL21 Kan^+^	[17]
9450	Tip adhesin MEFA in pET28α/BL21 Kan^+^	[17]
9331	His-tagged 3xSTa_N12S_-mnLT_R192G/L211A_, in pET28a/BL21 Kan^+^	[18]
9471	His-tag-less 3xSTa_N12S_-mnLT_R192G/L211A_, in pET28a/BL21 Kan^+^	[19]
9472	His-tag-less major subunit CFA MEFA, CFAI/II/IV in pET28a/BL21 Kan^+^	[19]
9175	His-tagged major subunit CFA MEFA CFAI/II/IV in pET28a/BL21 Kan^+^	[15]

### Mouse subcutaneous (SC) immunization with major subunit CFA/I/II/IV MEFA or tip adhesin MEFA

Five groups of eight-week old female BALB/c mice (Charles River Laboratories International, Inc., Wilmington, MA), nine mice per group, were included in subcutaneous (SC) immunization. The first group was administered with 17 μg (per mouse) of CFA/I/II/IV MEFA protein (9472) alone, the second was administered with 17 μg CFA/I/II/IV MEFA protein premixed with 20 μg toxoid fusion (9471), the third group was injected with 40 μg tip adhesin MEFA protein (9450) alone, and the fourth group was injected with 40 μg tip adhesin MEFA protein (9450) premixed with 20 μg toxoid fusion 3xSTa_N12S_-mnLT_R192G/L211A_ (9471). Based on protein molecular weight, 40 μg tip adhesin MEFA (~40 kDa) was calculated to carry equivalent molecule copy numbers as 17 μg major subunit CFA/I/II/IV MEFA (~17 kDa). One microgram holotoxin-structured double mutant heat-labile toxin, dmLT (LT_R192G/L211A_) provided by Walter Reed Army Institute of Research (Silver Spring, MD), was included as adjuvant for all four immunization groups (in the final volume of 40 μl, with antigen and adjuvant). The fifth group SC injected with 40 μl PBS was used as the control.

Two booster SC injections at the same doses of the primary were followed at the interval of two weeks. Blood samples were collected from each mouse before the primary immunization and two weeks after the final (second) booster. Mouse serum samples were stored at -80°C until use.

### Mouse subcutaneous immunization with ETEC CFA/I fimbriae, major structural subunit CfaB protein, or minor tip subunit CfaE protein

Three groups of eight-week-old female BALB/C mice, eight mice per group, were immunized with 40 μg CFA/I fimbriae heat-extracted from ETEC strain H10407, 40 μg CFA/I major subunit CfaB recombinant protein, or 40 μg CFA/I tip subunit CfaE recombinant protein, in the final volume of 40 μl. One μg dmLT adjuvant was included for three immunization groups. Immunized mice received two boosters at the dose of the primary, at the two-week interval. The fourth group of eight mice injected with PBS was used as the control. Mouse serum samples collected before the primary and after the second booster were stored at -80°C before use.

### Mouse serum anti-adhesin and antitoxin antibody titration

ELISAs with different coating antigens were used to titrate adhesin-specific IgG antibody responses from mouse serum samples. Since a typical CFA fimbria consists of hundreds or even thousands of copies of a major structural subunit but a few or even a single copy of the tip minor subunit, ELISAs using fimbriae as the coating antigen lead to no or low detection of tip-specific antibody responses induced by tip MEFA or a tip minor subunit. Therefore, in this study, heat-extracted fimbriae (CFA/I, CS1, CS2, CS3, CS4, CS5) and CS6 recombinant major subunit protein (CssA) were used as the ELISA coating antigens to titrate anti-adhesin IgG responses for the mice immunized with major subunit CFA/I/II/IV MEFA, CFA/I fimbriae or CFA/I major structural subunit CfaB; whereas recombinant tip minor subunit proteins were used to titrate antibodies for the mice immunized with tip MEFA or CFA/I tip minor subunit CfaE.

As described previously [15, 17], serum samples of the mice SC immunized with CFA/I/II/IV MEFA (9472), alone or combined with toxoid fusion 3xSTa_N12S_-mnLT_R192G/L211A_ (9471), were titrated for IgG antibodies to fimbriae CFA/I, CS1, CS2, CS3, CS4 and CS5, and to major subunit CssA of non-fimbrial CS6, respectively. Serum samples of the mice immunized with tip MEFA (with or without toxoid fusion 3xSTa_N12S_-mnLT_R192G/L211A_) were titrated for IgG responses to tip or adhesive subunit CfaE (CFA/I), CooD (CS1), CotD (CS2), CstH (CS3), CsaE (CS4), CsfD (CS5), CssB (CS6; adhesin and the other major structural subunit of CS6), LngA (CS21) and EtpA (EtpA). For the serum samples from the mice immunized with CFA/I fimbriae or CFA/I major subunit protein CfaB, anti-CfaB or anti-CFA/I response was titrated in ELISAs with recombinant protein CfaB (or CFA/I fimbriae) as the coating antigen. To titrate IgG response to CfaE tip subunit from the serum samples of the mice immunized with CFA/I fimbriae or CfaE tip subunit, CfaE recombinant protein was used accordingly as the ELISA coating antigen. Briefly, 400 ng heat-extracted adhesin fimbriae or each major or minor subunit recombinant protein was coated to each well of 2HB plates (Thermo Scientific, Rochester, NY) at 4°C overnight. Coated plates were washed with PBST (PBS with 0.05% tween-20), treated with 5% fat-free milk-PBST to block uncoated sites, then washed with PBST, and incubated with serum samples (twofold dilutions, from 1:200 to 1:25,600) from each immunized or control mouse at 37°C for 1h. Horseradish peroxidase (HRP)-conjugated goat anti-mouse IgG (1:3300; Sigma) and 3,3’,5,5’-tetramethylbenzidine (TMB) Microwell Peroxidase Substrate System (2-C) (KPL, Gaithersburg, MD) were used to measure optical density OD_650_. Antibody titers were calculated from the highest dilution of a serum sample that produced OD readings of > 0.3 (after subtraction of the background readings; dilution multiplied by adjusted OD) and were presented in log_10_ scale as previously described [15, 17].

Serum samples of the mice SC immunized with major subunit CFA/I/II/IV MEFA or tip MEFA combined with toxoid fusion 3xSTa_N12S_-mnLT_R192G/L211A_ were also titrated for IgG antibodies specific to LT and STa. One hundred ng CT (per well of 2HB plates) or 10 ng STa-ovalbumin conjugates (per well of Costar plates; Corning Inc., Corning, NY) was used as the ELISA coating antigen as described previously [18, 20, 21].

### Mouse serum anti-adhesin antibody adherence inhibition assays

Mouse serum samples pooled from each immunization group or the control group were examined for *in vitro* antibody adherence inhibition against ETEC or *E*. *coli* bacteria expressing CFA/I, CS1, CS2, CS3, CS4/CS6, CS5/CS6, CS6, CS21 and *EtpA*, using Caco-2 cells (ATCC, #HTB-37), as previously described [15, 17]. Briefly, ETEC or *E*. *coli* bacteria (1.5x10^6^ per well) expressing each target adhesin (which were pre-verified for adherence to Caco-2 cells) were mixed with 10% mannose and then 20 μl mouse serum samples from each group; each mixture was added to 95% confluent monolayer Caco-2 cells (1.5 x10^5^ per well). Incubated in a CO_2_ incubator (5% CO_2_) for 1 h at 37°C, Caco-2 cells were gently washed with PBS to remove non-adherent ETEC or *E*. *coli* bacteria, dislodged with 0.5% Triton X-100 (Sigma, St. Louis, MO), collected with centrifugation (15,000 g for 10 min), and then suspended in 1 ml PBS. Bacteria suspensions were serially diluted and plated on LB plates. Overnight grown ETEC or *E*. *coli* bacteria (CFUs) were counted. Additionally, *E*. *coli* strain DH5α that does not express specific ETEC fimbriae or adhesins was included to assess if any antibodies induced by potential protein contaminants including LPS inhibited bacteria adherence non-specifically.

### Antigen tolerance and histopathology microscopic examination

Liver, spleen, kidney, intestine, and skin and subcutaneous fat tissues at injection sites were collected from each group of the mice SC immunized with major subunit CFA/I/II/IV MEFA, the tip MEFA, alone or combined with toxoid fusion 3xSTa_N12S_-mnLT_R192G/L211A_, and the control mice respectively at necropsy. Tissue samples were immediately fixed in 10% neutral buffered formalin (Valley Vet Supply, Marysville, KS). Fixed tissues were processed in an automated tissue processor, embedded in paraffin, and sectioned. Slide-mounted tissue sections were stained with hematoxylin and eosin (H&E), and blind-labeled sections were examined microscopically by a board-certified pathologist. Microscopic lesions were estimated based on the following scoring system: 0, no inflammation (normal); 1, small aggregates and perivascular cuffs of plasma cells and/or macrophages, inflammation is localized and not readily apparent; 2, multiple, small aggregates of plasma cells and lymphocytes and macrophages, with and without neutrophils, that are beginning to coalesce; 3, the inflammation is similar but is coalescing and forms small plaque-like nodules in the subcutaneous fat.

### Mouse immunization and challenge studies

Six groups of eight-week old female CD-1 (ICR) mice (Charles River Laboratories), eight mice per group, were used for mouse immunization and challenge studies. Among them, four groups were SC immunized with extracted CFA/I fimbriae (40 μg), major subunit CfaB recombinant protein (40 μg), tip adhesin subunit CfaE recombinant protein (40 μg), or PBS (40 μl) respectively. These immunized mice received two boosters at the same dose of the primary at the two-week interval. Mice in the fifth group were primed with oral gavage of H10407 (5x10^4^ CFUs), followed by two SC injections of CFA/I fimbriae (40 μg). The sixth group received prime oral inoculation of H10407 (5x10^4^ CFUs) only.

Mouse ileum colonization study followed a published protocol [22]. Briefly, mice received streptomycin (5 g/L) and fructose (6.7%) in the drinking water for 24 h prior to challenge to eradicate facultative anaerobes, followed by sterile water without food for 12 hours. Famotidine (50mg/kg) was administered 2 h prior to challenge to neutralize gastric acid. Mice were subsequently challenged by gavage of 200 μl of inoculum containing ETEC H10407 strain (5x10^3^ CFU). Mice were sacrificed 24 h post inoculation, with food was withdrawn 12 prior to euthanasia. Two sections of ileum (three cm in length) were collected, followed by vortex for 10 seconds and incubation in triton X-100 (0.5%) for 10 min. Ileum lysates were diluted in PBS and plated on LB plates. Overnight grown colonies (CFUs) were counted. Fifty colonies randomly selected from each group were verified in PCR with primers specific to STa gene (Forward 5’- TAATGTTGGCAATTTTTATTTCTGT-3’; Reverse 5’- ATCCAGCACAGGCAGGATTA-3’).

All mouse studies complied with the 1996 National Research Council guidelines and were approved and supervised by Kansas State University Institutional Animal Care and Use committee (IACUC).

### Statistical analysis

Data from mouse serum IgG antibody titration (titers normalized in log_10_), *in vitro* antibody adherence inhibition assay, and mouse ileum colonization study were examined for differences using SAS 8 (version 8; SAS Institute, Cary, NC) or GraphPad prism software (version 7.0; La Jolla, CA). Antibody titration and mouse ileum colonization study were carried out twice, and data were normalized by log transformation and then compared for differences between different treatment groups using One-way ANOVA with Tukey’s post hoc test at 95% confidence. *In vitro* antibody adherence inhibition assays were repeated twice, and data were analyzed using Kruskal-Wallis test, followed by Dunn’s pairwise comparison. Differences were considered significant if a *p* value was less than 0.05.

## Results

### Mice subcutaneously immunized with major subunit CFA/I/II/IV MEFA alone or combined with toxoid fusion 3xSTa_N12S_-mnLT_R192G/L211A_ developed antibody responses to all seven ETEC adhesins

**Fig 1:**
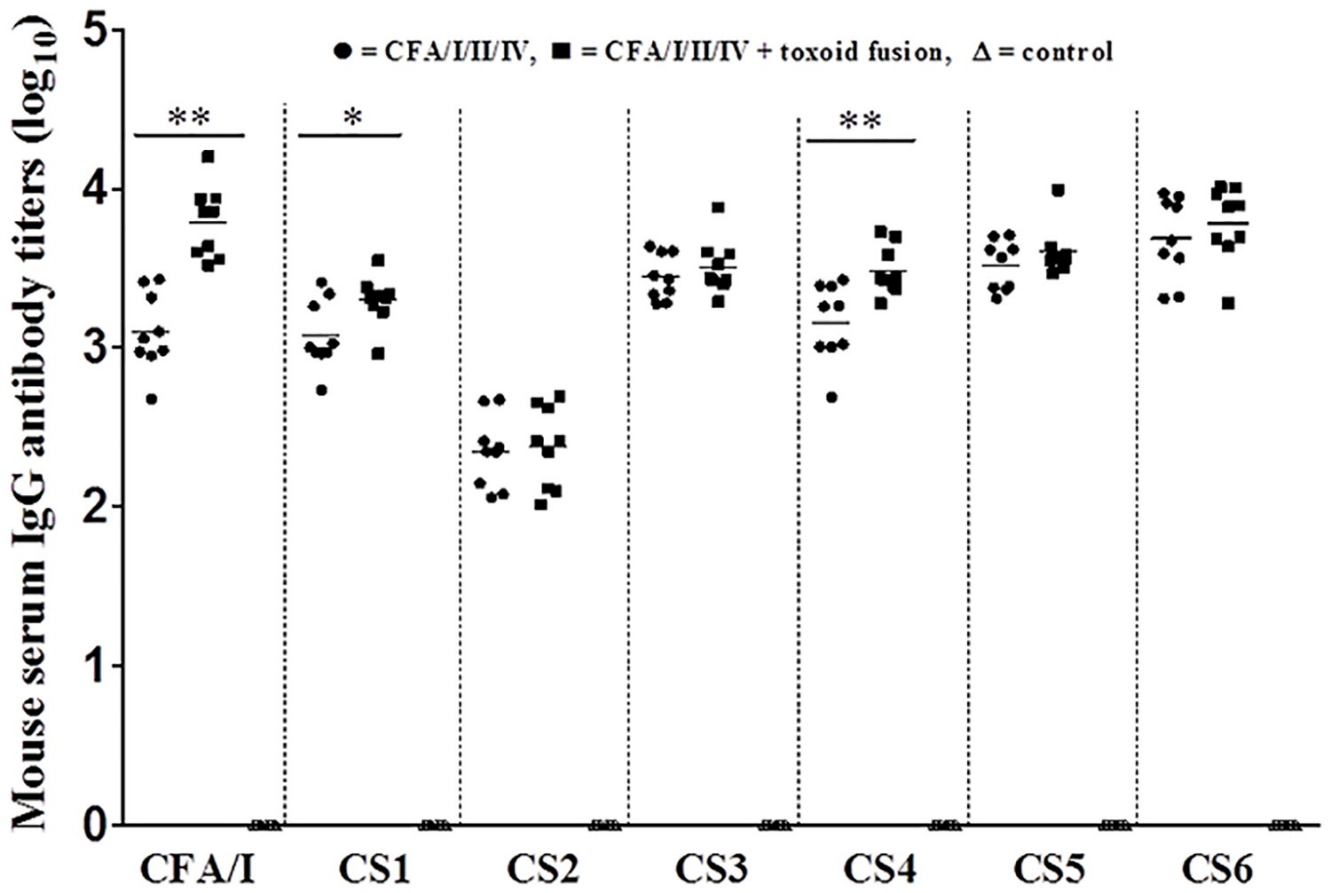
Serum anti-CFA/I, -CS1, -CS2, -CS3, -CS4, -CS5 and anti-CS6 (CssA) IgG antibody titers (log_10_) in the mice subcutaneously immunized with major subunit CFA/I/II/IV MEFA (●), major subunit CFA/I/II/IV MEFA combined with toxoid fusion 3xSTa_N12S_-mnLT_R192G/L211A_ (■), or the control mice (Δ; no response detected). Antibody titers were compared for differences between different treatment groups using One-way ANOVA with Tukey’s post hoc test. Bars represent the mean titers of each group. Each dot represents an antigen-specific titer from a mouse in the group. * indicates a p-value of <0.05, and ** shows a p-value of <0.01.

Mice SC immunized with major subunit CFA/I/II/IV MEFA alone or the major subunit MEFA premixed with toxoid fusion 3xSTa_N12S_-mnLT_R192G/L211A_ developed anti-adhesin IgG antibody responses (Fig 1). Mouse serum anti-CFA/I, -CS1, -CS2, -CS3, -CS4, -CS5, and anti-CS6 IgG titers were detected at 3.1 ± 0.23, 3.1 ± 0.20, 2.3 ± 0.21, 3.4 ± 0.13, 3.2 ± 0.23, 3.5 ± 0.15 and 3.7 ± 0.25 (log_10_) in the group immunized with major subunit CFA/I/II/IV MEFA alone, and 3.8 ± 0.21, 3.3 ± 0.15, 2.4 ± 0.24, 3.5 ± 0.16, 3.5 ± 0.14, 3.6 ± 0.14, and 3.8 ± 0.22 (log10) in the group co-immunized with the major subunit MEFA and the toxoid fusion. Mice co-immunized with CFA/I/II/IV MEFA and toxoid fusion 3xSTa_N12S_-mnLT_R192G/L211A_ developed greater IgG titers, with IgG titers specific to CFA/I (p<0.01), CS1 (p<0.05) and CS4 (p<0.01) were significantly greater compared to those in the mice immunized with major subunit CFA/I/II/IV MEFA only (Fig 1).

Anti-LT (2.7 ± 0.35) and anti-STa (2.7 ± 0.35) IgG titers were detected in the serum samples of mice co-immunized with CFA/I/II/IV MEFA and toxoid fusion 3xSTa_N12S_-mnLT_R192G/L211A_. No anti-adhesin or antitoxin IgG antibody response was detected from the control serum samples.

### Mice SC immunized with tip MEFA, with or without toxoid fusion 3xSTa_N12S_-mnLT_R192G/L211A_, developed similar levels of antibody responses to all nine CFA tip subunits or adhesive subunits

**Fig 2:**
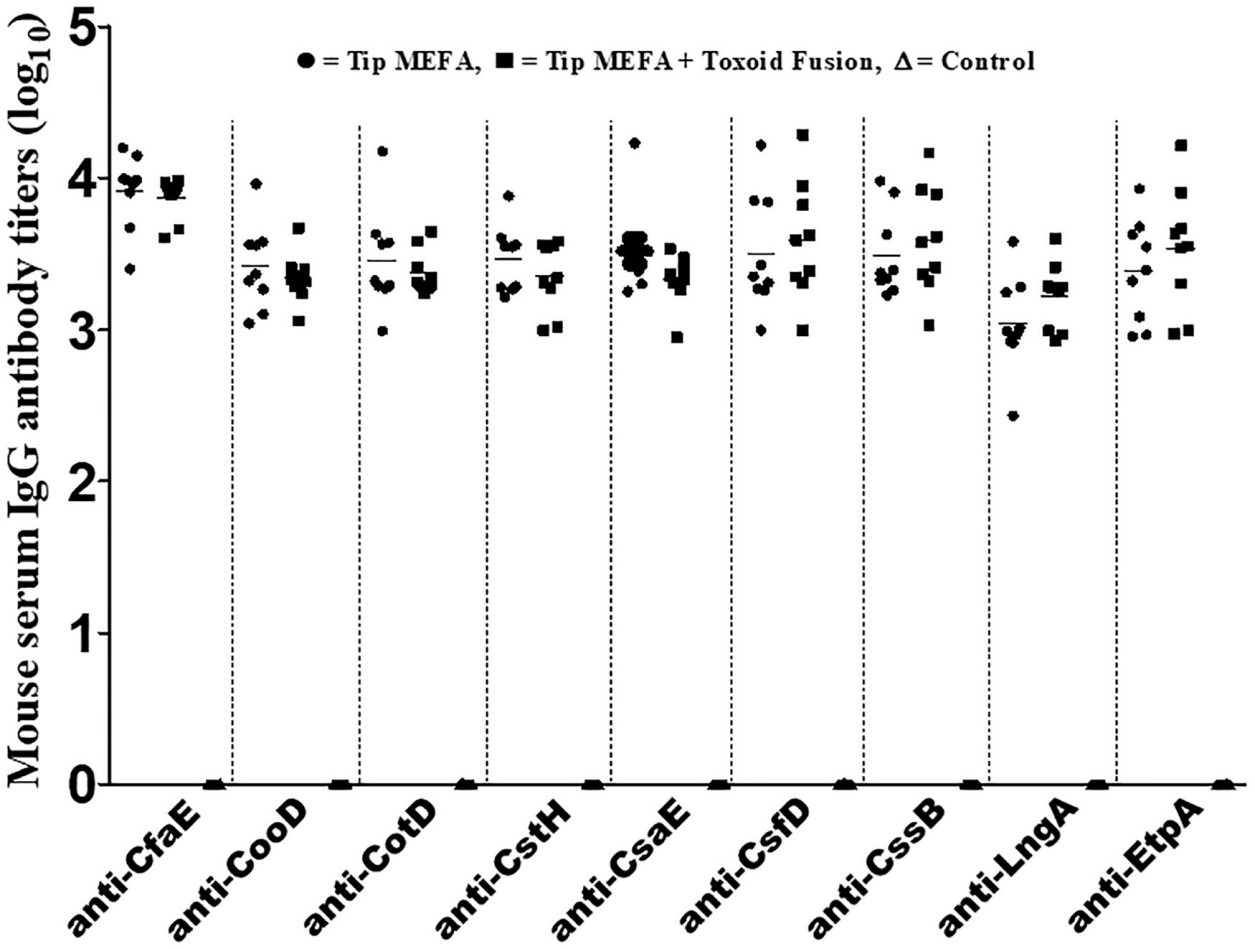
Serum anti-CfaE (CFA/I), -CooD (CS1), -CotD (CS2), -CstH (CS3), -CsaE (CS4), -CsfD (CS5), -CssB (CS6), -LngA (CS21) and anti-EtpA IgG antibody titers (log_10_) in the mice subcutaneously immunized with tip MEFA (●), tip MEFA combined with toxoid fusion 3xSTa_N12S_-mnLT_R192G/L211A_ (■), or the control mice (Δ; no response detected). Titers (in log_10_) were compared for differences between two immunization groups by using One-way ANOVA with Tukey’s post hoc test. Bars indicate the mean titers of each group. Each dot indicates a titer specific to each tip adhesin from a mouse.

Serum samples of the mice SC immunized with tip MEFA alone or tip MEFA combined with toxoid fusion 3xSTa_N12S_-mnLT_R192G/L211A_ developed IgG antibody responses to all target tip subunits or adhesive subunits (Fig 2; recombinant tip or adhesive subunit proteins as the ELISA coating antigens). Anti-CfaE (CFA/I), -CooD (CS1), -CotD (CS2), -CstH (CS3), -CsaE (CS4), -CsfD (CS5), -CssB (CS6), -LngA (CS21) and anti-EtpA IgG titers were detected at 3.9 ± 0.23, 3.4 ± 0.26, 3.5 ± 0.31, 3.5 ± 0.20, 3.5 ± 0.27, 3.5 ± 0.36, 3.5 ± 0.26, 3.0 ± 0.29 and 3.4 ± 0.31 (in log_10_) in the serum of the mice immunized with tip MEFA alone; whereas the titers were 3.9 ± 0.12, 3.3 ± 0.15, 3.4 ± 0.13, 3.4 ± 0.21, 3.3 ± 0.15, 3.6 ± 0.36, 3.6 ± 0.33, 3.2 ± 0.20 and 3.5 ± 0.37 (in log_10_) in the serum of the mice co-immunized with tip MEFA and toxoid fusion 3xSTa_N12S_-mnLT_R192G/L211A_. Anti-mouse IgG titers specific to each tip subunit or adhesive subunit from these two immunization groups were not significantly different (Fig 2).

Anti-LT and anti-STa IgG titers were detected at 2.6 ± 0.28 and 2.3 ± 0.27 (log_10_) respectively in the serum samples of mice co-immunized with tip MEFA and toxoid fusion 3xSTa_N12S_-mnLT_R192G/L211A_. No antibody to STa, LT, or any of the target tip subunits was detected in the control group.

IgA antibody responses in mouse serum or fecal samples were not included in this study due to the low and inconsistent titers from initial analyses.

### Serum antibodies from the mice SC immunized with major subunit CFA/I/II/IV MEFA or tip MEFA, with or without the toxoid fusion, significantly inhibited adherence of *E*. *coli* or ETEC bacteria expressing representing adhesins to Caco-2 cells

**Fig 3:**
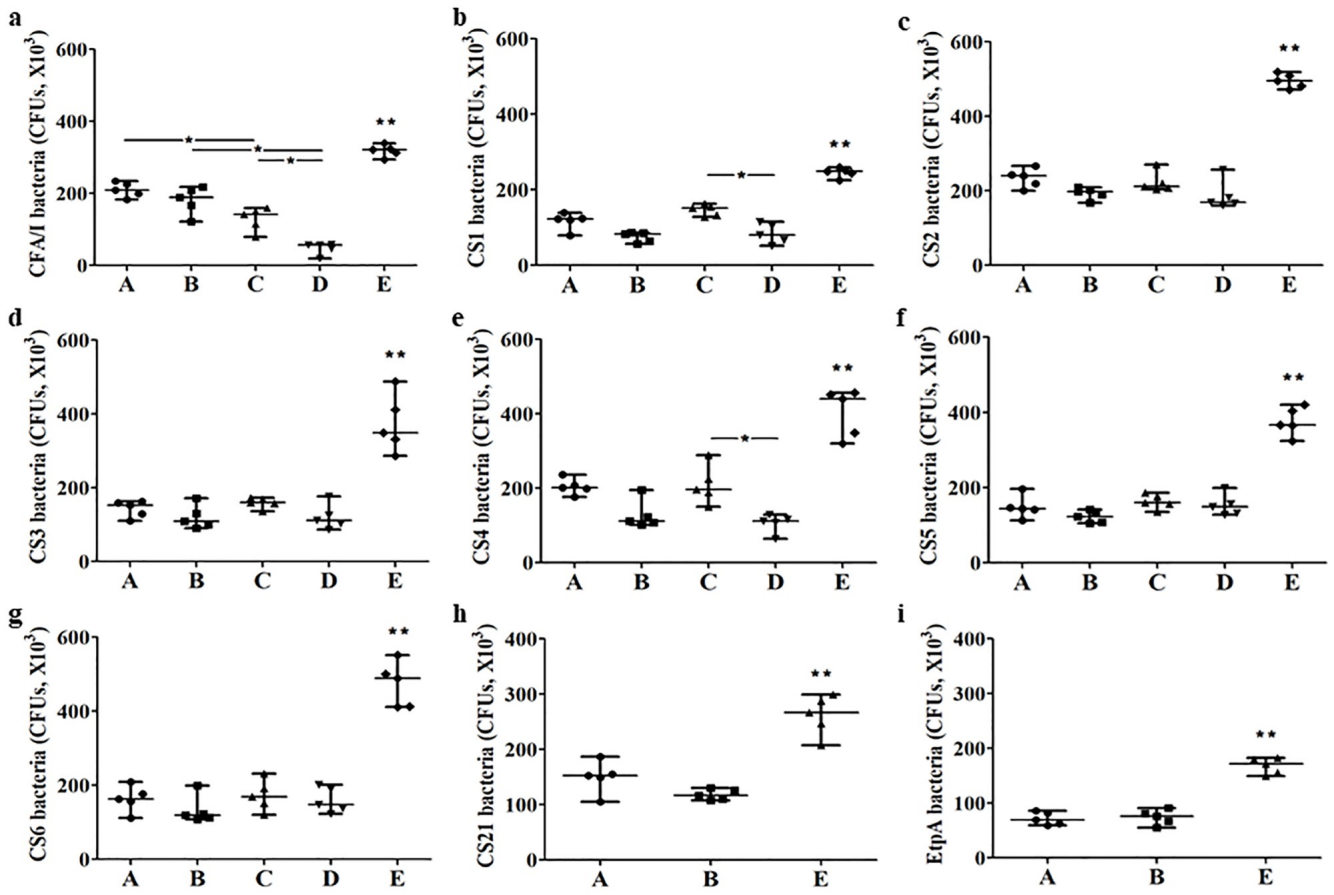
*In vitro* mouse serum antibody adherence inhibition assays from the groups subcutaneously immunized with tip MEFA alone (A), tip MEFA combined with toxoid fusion 3xSTa_N12S_-mnLT_R192G/L211A_ (B), major subunit CFA/I/II/IV MEFA alone (C), major subunit CFA/I/II/IV MEFA combined with toxoid fusion 3xSTa_N12S_-mnLT_R192G/L211A_ (D), or the control group (E). The line in each group represents the median number of each bacteria expressing CFA/I (panel a), CS1 (panel b), CS2 (panel c), CS3 (panel d), CS4/CS6 (panel e), CS5/CS6 (panel f), CS6 (panel g), CS21 (panel h) or EtpA (panel i) adhesin (CFUs) adherent to Caco-2 cells. Data were analyzed using Kruskal-Wallis test, followed by Dunn’s pairwise comparison. * indicates a p-value of <0.05, and ** refers a p-value of <0.01 (comparison between the control group and any of the immunization groups).

*E*. *coli* or ETEC bacteria expressing CFA/I (H10407), CS1 (THK38/pEU405), CS2 (DH5a/pEU588), CS3 (E116), CS4/CS6 (E106), CS5/CS6 (UM75688), or CS6 (JF2423), after incubation with the pooled mouse serum of the group SC immunized with major subunit CFA/I/II/IV MEFA or tip MEFA, with or without the co-administer of toxoid fusion 3xSTa_N12S_-mnLT_R192G/L211A_, showed a significant reduction in adherence to Caco-2 cells, compared to those incubated with the control mouse serum (Fig 3a–3g). ETEC bacteria expressing CS21 (JF2101) or EtpA adhesin (JF2318) were also shown a significant reduction in adherence to Caco-2 cells, after incubation with the serum of the group SC immunized with tip MEFA, alone or combined with the toxoid fusion (Fig 3h and 3i).

*E*. *coli* or ETEC bacteria expressing CS1, CS2, CS3, CS4/CS6, CS5/CS6 or CS6 adhesins showed similar levels of reduction in adherence (to Caco-2 cells) after incubation with the serum from the mice SC immunized with major subunit CFA/I/II/IV MEFA or tip MEFA. Only ETEC H10407 had a significantly more reduction in adherence to Caco-2 cells after incubation with the serum of the mice immunized with the major subunit MEFA, compared to the serum from the mice immunized with tip MEFA regardless co-administration of the toxoid fusion antigen (p<0.05; Fig 3a).

No significant differences were shown from bacterial adherence inhibition using the serum from the mice immunized with tip MEFA alone compared to the serum from the mice co-immunized with tip MEFA and toxoid fusion 3xSTa_N12S_—mnLT_R192G/L211A_. Comparatively, the serum from the mice co-immunized with major subunit CFA/I/II/IV MEFA and the toxoid fusion showed an increasing reduction against adherence of bacteria expressing CFA/I, CS1 or CS4/CS6, compared to the serum from the mice immunized with CFA/I/II/IV MEFA alone (Fig 3a, 3b and 3e).

### Tip MEFA and major subunit CFA/I/II/IV MEFA, alone or combined with toxoid fusion 3xSTa_N12S_-mnLT_R192G/L211A_, were well tolerated in the SC administered mice

Mice tolerated well to subcutaneously administered tip MEFA, major subunit CFA/I/II/IV MEFA, and toxoid fusion 3xSTa_N12S_-mnLT_R192G/L211A_ recombinant proteins. No gross lesions were found from tissues of liver, spleen, kidney, intestine or skin at the injection sites of the immunized mice (lesion score = 0). Microscopic examination showed there were no visible differences between tissues of any immunized mice and tissues of the control mice. Not-readily-apparent or mild localized inflammation with small aggregates and perivascular cuffs of plasma cells and/or macrophages were found at the skin (site of injection) of the mice immunized with tip MEFA alone or tip MEFA combined with the toxoid fusion (Panels A or B in S1 Fig). Only small aggregates of plasma cells and lymphocytes and macrophages, with and without neutrophils, were observed from the skin (the injection site) of the mice immunized with major subunit CFA/I/II/IV MEFA or major subunit CFA/I/II/IV MEFA together with the toxoid fusion. Observed inflammation was coalescing, which formed small plaque-like nodules in the subcutaneous fat (Panels C or D in S1 Fig).

### Serum samples of the mice SC immunized with CFA/I fimbriae, CfaB major subunit, or CfaE tip subunit inhibited adherence of ETEC H10407

**Fig 4:**
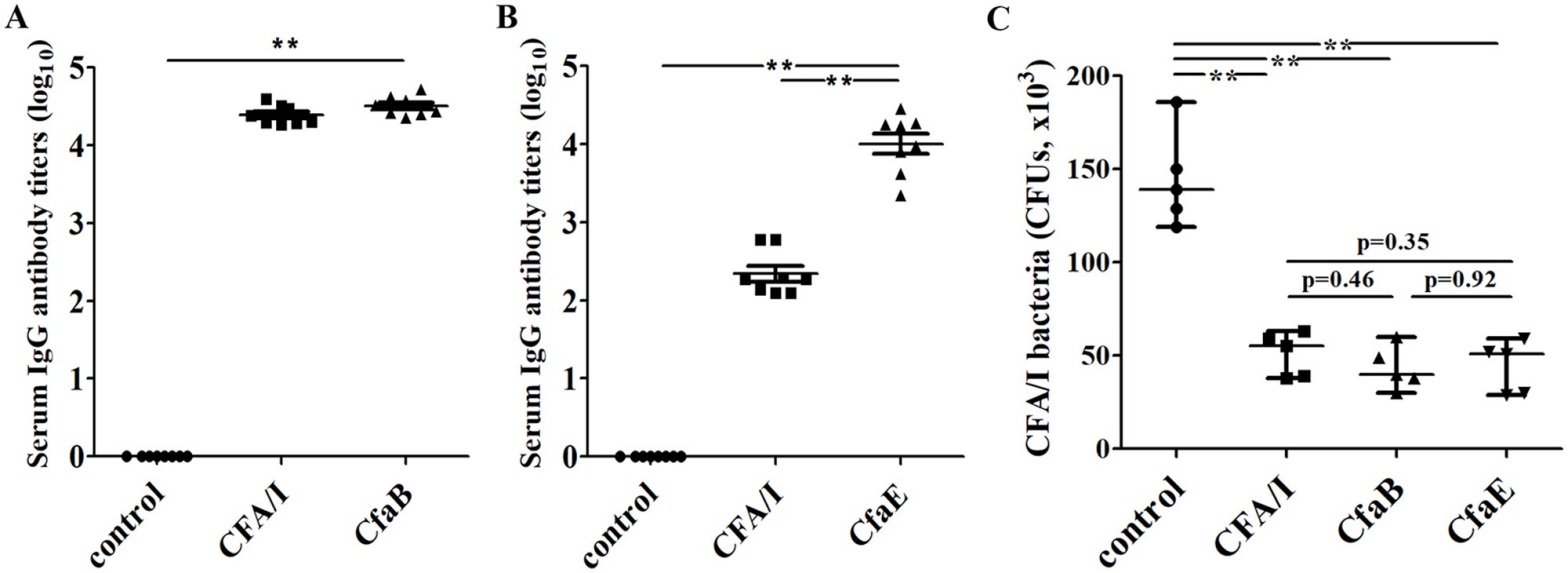
Mouse serum antibody titers (log_10_) and *in vitro* antibody adherence inhibition against ETEC H10407 (LT/STa/CFA/I). Panel A: anti-CfaB IgG titers from the mice subcutaneously immunized with CFA/I fimbriae or CfaB major subunit recombinant protein, or the control mice; with major subunit CfaB recombinant protein as the ELISA coating antigen. Panel B: anti-CfaE IgG titers from the mice subcutaneously immunized with CFA/I fimbriae or CfaE tip subunit protein, or the control mice; with CFA/I tip subunit CfaE recombinant protein as the ELISA coating antigen. Panel C: antibody adherence inhibition against ETEC H10407 bacteria (1x10^3^ CFUs) to Caco-2 cells from the serum samples of the mice immunized with CFA/I fimbriae, CFA/I major subunit CfaB, or CFA/I tip subunit CfaE. Antibody titers (A and B) and antibody inhibition data (C) were compared for differences between groups using One-way ANOVA with Tukey’s post hoc test and Kruskal-Wallis test with Dunn’s pairwise comparison, respectively. The line in each group represents the mean with standard deviation (A and B) or median with range (C). ** indicates a p-value of <0.01, compared the control with each immunization group.

Mice SC immunized with CFA/I fimbriae or CFA/I major subunit CfaB recombinant protein developed similar levels of IgG antibody titers to CfaB (Fig 4A; recombinant CfaB protein as the ELISA coating antigen) or CFA/I fimbriae (heat-extracted CFA/I fimbriae as the ELISA coating antigen). The mice immunized with CFA/I fimbriae or tip subunit CfaE protein developed IgG antibodies to CfaE (Fig 4B; recombinant CfaE protein as the ELISA coating antigen). No anti-CfaB or anti-CfaE antibody response was detected from the control mouse serum samples.

Mouse serum samples from the groups immunized with CFA/I fimbriae, major subunit CfaB, or tip subunit CfaE showed similar levels of antibody inhibition activity against adherence of CFA/I fimbrial H10407 ETEC bacteria to Caco-2 cells (Fig 4C). The medians of H10407 bacteria (CFUs; x10^3^) adherent to Caco-2 cells were 55, 40, and 51 with ranges 38–63, 30–60, and 29–59 after incubated with the serum samples of the mice SC immunized with CFA/I fimbriae, CfaB major subunit protein, or CfaE minor tip adhesin subunit protein. No significant differences were observed at the numbers of bacteria adherent to Caco-2 cells after incubation with the serum of the immunized groups (Fig 4C). The median adherent H10407 bacteria at Caco-2 cells incubated with the serum samples of the control mice was 139 (range 119–186, CFUs; x10^3^), significantly greater than those treated with the serum of each immunization group (p<0.01).

### Mice SC immunized with CFA/I fimbriae, CfaB major subunit, or CfaE tip subunit had fewer bacteria colonized after challenge with ETEC strain H10407

**Fig 5:**
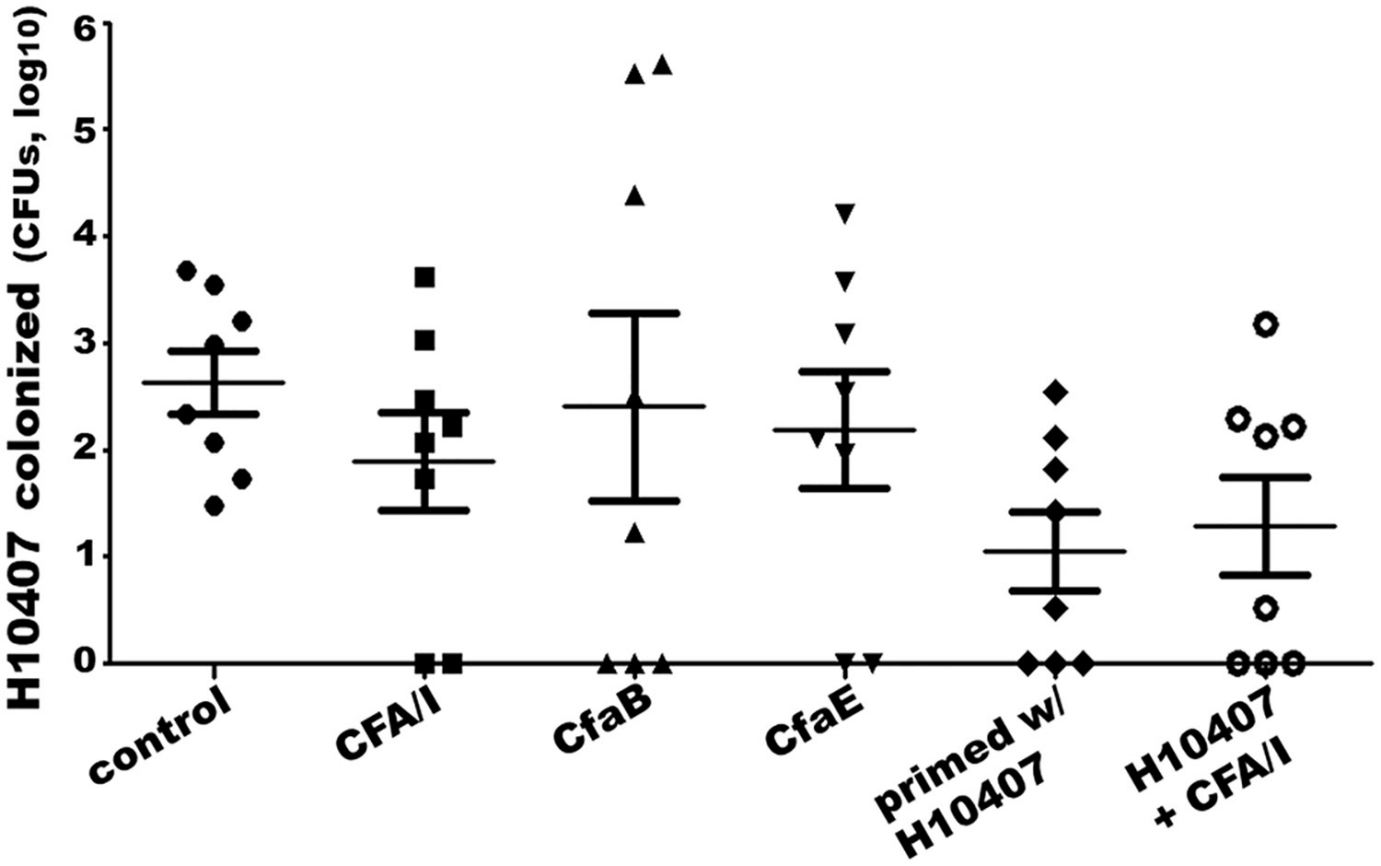
Bacteria colonization from mouse ileum samples after challenge with ETEC strain H10407. Mice were challenged with H10407 after SC immunized with PBS (control), heat-extracted CFA/I fimbria (CFA/I), recombinant major subunit CfaB (CfaB), or recombinant tip subunit CfaE (CfaE), orally inoculated with H10407 (primed w/H10407), or orally primed with H10407 followed with SC boosters with CFA/I fimbriae (H10407 + CFA/I). Numbers of H10407 bacteria collected from mouse ileum samples were cultured and counted (CFUs in log_10_). Data were normalized by log transformation and analyzed using One-way ANOVA with Tukey’s post hoc test. The line in each group represents the mean with standard deviation.

Mice SC immunized with CFA/I fimbriae, CfaB major subunit recombinant protein, or CfaE tip adhesin subunit protein showed fewer bacteria colonized in mouse intestines after orally inoculation of ETEC strain H10407. However, the reduction of the H10407 bacteria was not statistically significant between each immunized group and the control group (Fig 5).

Additionally, two groups of mice immunized (or challenged) with gavage inoculation of live H10407 bacteria, with or without boosters of SC-administered CFA/I fimbriae, also developed IgG antibodies to CFA/I. These mice showed fewer bacteria colonized when they were re-challenged with H10407 (Fig 5). However, reduction of bacteria colonization was not significant compared with the control group, or any of the groups SC immunized with CFA/I fimbriae, major subunit CfaB, or tip subunit CfaE (Fig 5).

## Discussion

Data from the current study indicated that antibodies derived from ETEC major structural subunits and minor tip subunits, or epitopes of a major subunit and a minor tip subunit, appear to be equivalently effective against bacteria adherence *in vitro*. Since ETEC tip subunits or adhesive subunits play an essential role in bacterial inter-reaction with host cell receptors, anti-tip subunit antibodies which directly block attachment of ETEC fimbriae to host cell receptors can effectively protect against ETEC bacterial colonization and thus diarrheal disease. Antibodies against a fimbrial major structural subunit, unless this major subunit is also the adhesive subunit which is directly involved in bacterial adherence, do not serve as a direct blockage to prevent the recognition between a fimbria and host receptors. A recent study, however, demonstrated that anti-fimbria antibodies, particularly antibodies to ETEC major structural subunits can physically damage fimbria adherence function [23]. Anti-fimbria or anti-major subunit antibodies recognize adjacent major subunits and lock extension and contraction movement of coil-structured fimbriae. That makes fimbria “stiff and tangled”, resulting in reduction or prevention of ETEC fimbria adherence to host cells [23]. Since the molecular sizes of IgG and more particularly IgA antibodies to CFA major subunits are much larger compared to a typical ETEC tip subunit (15 to 50 kDa), anti-major subunit antibodies recognized the major structural subunits adjacent to the tip subunit can physically prevent a tip from adhering to host receptors (S2 Fig). The high copy number of a major subunit in an ETEC fimbria also intensify the interaction between anti-major-subunit antibodies and fimbriae. Thus, anti-major subunit antibodies can be equivalently effective as anti-tip antibodies to inhibit fimbrial adherence to host cells.

Results from the comparative study of antibody adherence inhibition showed antibodies induced by CFA/I fimbriae, major structural subunit CfaB or tip subunit CfaE were equally effective at blocking the adherence of ETEC H10407 bacteria to Caco-2 cells. This directly supported the notion that antibodies derived from an ETEC major structural subunit or a tip subunit can be equivalently effective against ETEC bacterial adherence. Mice immunized with CFA/I fimbriae or CfaB major subunit developed similar titers of anti-CfaB IgG antibodies. This is not a surprise because CFA/I fimbria is largely composed of CfaB major subunits. In contrast, mice immunized with CfaE tip subunit protein developed significantly greater anti-CfaE IgG response compared to mice immunized with CFA/I fimbriae (Fig 4B), directly resulted from a low copy number of CfaE tip subunit molecule carried by CFA/I fimbria. With the same amount (40 μg) of CFA/I fimbriae and recombinant CfaE protein used for immunization, the mice administered with CfaE protein received a significantly higher dose of CfaE antigen, thus developed greater titers of anti-CfaE antibodies (Fig 4B).

It was observed that the mice SC immunized with CFA/I fimbrial adhesin, major subunit CfaB, or tip adhesin CfaE, or mice gavage inoculation with H10407 regardless the booster of SC immunization with CFA/I fimbriae, showed a reduction in colonization by ETEC challenge strain H10407. It was also noted that challenge H10407 bacteria was not recovered from the ileum tissue of some individual mice only in the immunization groups (but not the control group) (Fig 5). However, the overall reduction of H10407 colonized in ileum of the immunized mice was not significant compared to the control mice. Insignificance against H10407 colonization could be resulted from the small sampling size or a need of further improved mouse challenge model. Future studies with suitable animal models including a rabbit colonization model or a controlled human infection model (CHIM) should evaluate better antibodies derived from fimbrial subunits against ETEC colonization and diarrheal disease.

We recently demonstrated that ETEC fimbrial subunit epitopes (instead of an entire subunit) presented by a backbone subunit protein can induce antibodies against ETEC bacteria adherence. CFA/I/II/IV MEFA (multiepitope fusion antigen) that uses CFA/I major subunit CfaB backbone to carry representative epitopes from major structural subunits of CS1 to CS6 adhesins, and tip MEFA with CFA/I minor tip subunit CfaE backbone to carry epitopes from minor tip subunits (or adhesive subunits) induced antibodies inhibiting adherence of target ETEC bacteria [15, 17]. In the current study, we subcutaneously immunized mice with equivalent molecules of two MEFA proteins and found that major subunit CFA/I/II/IV MEFA and tip MEFA induced antibodies with a similar level of activity against bacteria adherence. *In vitro* antibody adherence inhibition assays indicated that antibodies derived from two MEFAs (major subunit CFA/I/II/IV MEFA versus tip MEFA) exhibited similar levels of inhibition activities against CS1 to CS6 adherence, suggesting the major subunit epitopes and the minor tip epitopes from these six adhesins are equivalently effective in inducing neutralizing anti-adhesin antibodies. However, major subunit CFA/I/II/IV MEFA induced antibodies showed greater inhibition activity against adherence of CFA/I fimbrial strain H10407 (Fig 3a). Future studies to examine if the epitopes of CFA/I/II/IV MEFA backbone (CfaB) are more neutralizing than the epitopes of tip adhesin MEFA backbone (CfaE) may provide insight regarding inhibition activity of derived-antibodies against CFA/I adherence.

Co-administration with toxoid fusion 3xSTa_N12S_-mnLT_R192G/L211A_ did not alter immunogenicity of tip MEFA. Tip MEFA when subcutaneously immunized alone or together with the toxoid fusion induced similar levels of IgG antibody responses to all nine tip adhesins. Moreover, mouse serum antibodies showed no differences at adherence inhibition activities against these nine ETEC adhesins. Noticeably, co-administer with toxoid fusion 3xSTa_N12S_-mnLT_R192G/L211A_ enhanced major subunit CFA/I/II/IV MEFA in inducing anti-CFA/I, anti-CS1 and anti-CS4 (but not to CS2, CS3, CS5 and CS6) IgG antibodies. Consequently, antibodies derived from co-administered CFA/I/II/IV MEFA and toxoid fusion 3xSTa_N12S_-mnLT_R192G/L211A_ showed greater adherence inhibition activities against CFA/I, CS1, and CS4 adhesins. How the co-administered toxoid fusion enhanced immunogenicity of CFA/I/II/IV MEFA is unclear momently. Nevertheless, data from this study indicated that the co-administer with the toxoid fusion had no negative impact to the immunogenicity of major subunit MEFA or tip MEFA. This provides very important information for ETEC vaccine development, because an ideal ETEC vaccine likely needs to include adhesin and toxin (toxoid) antigens to induce anti-adhesin antibodies as well as antitoxin antibodies for broad protection against ETEC diarrhea.

Additionally, histological microscopic results from this study revealed that neither tip MEFA or major subunit CFA/I/II/IV MEFA, when were subcutaneously administered alone or together with toxoid fusion 3xSTa_N12S_-mnLT_R192G/L211A_, did not cause any noticeable gross lesions in mouse internal organs or skin at the injection site. That indicated that all three antigens were well tolerated, suggesting either major subunit CFA/I/II/IV MEFA or tip MEFA can be combined safely with toxoid fusion 3xSTa_N12S_-mnLT_R192G/L211A_ as multivalent antigens for an ETEC vaccine, thorough detailed toxicology studies will be needed before we can proceed to human volunteer studies with these antigens.

Data from this study also showed that serum antibodies from the mice immunized with dmLT adjuvant or major subunit CFA/I/II/IV MEFA together with dmLT adjuvant had no inhibition activity against adherence of *E*. *coli* strain DH5α which does not express ETEC fimbriae (S3 Fig). That clearly indicated that adherence inhibition activities from antibodies induced by CFA/I/II/IV MEFA, tip MEFA, CFA/I fimbriae, or CFA/I major subunit CfaB or tip subunit CfaE are specific to adhesins.

In conclusion, data from this study suggest a fimbrial major structural subunit and a minor tip subunit were effective antigens in inducing anti-adhesin antibodies against ETEC bacterial adherence. Data also indicated that ETEC tip MEFA and major subunit CFA/I/II/IV MEFA can be candidate antigens for an ETEC anti-adhesin vaccine. Results showed both MEFAs can be safely co-administered with toxoid fusion 3xSTa_N12S_-mnLT_R192G/L211A_ which has already demonstrated to induce protective antitoxin antibodies against LT and STa enterotoxicity but also diarrhea in a pig challenge model [24], suggesting either MEFA can be combined with the toxoid fusion for the development of a broadly protective vaccine against ETEC diarrhea. Future studies using other immunization routes, including intramuscular (IM) and intradermal (ID), and examining anti-adhesin IgG and surely IgA antibodies for protection against *in vitro* and *in vivo* bacterial adherence, and more importantly against ETEC diarrhea will be needed. Data from these studies can help us to further verify immunogenicity of these two MEFAs and to evaluate better the candidacy of either MEFA for developing an effective vaccine against ETEC diarrhea.

## Supporting information

S1 Fig H&E-stained histological microscopy images to show not readily apparent localized inflammation at the skin injection sites of the subcutaneously immunized mice, on 10^th^ day after the second booster. **A**: skin of a mouse SC immunized with the tip adhesin MEFA (9450) alone. **B**: skin of mice SC immunized with tip adhesin MEFA (9450) combined with toxoid fusion 3xSTa_N12S_-mnLT_R192G/L211A_ (9471). Both images are showing not readily apparent localized inflammation. **C**: skin of a mouse SC immunized with the major subunit CFA/I/II/IV MEFA (9472) alone. **D**: skin of mice SC immunized with the major subunit CFA/I/II/IV MEFA (9472) combined with toxoid fusion 3xSTa_N12S_-mnLT_R192G/L211A_ (9471). The inflammation is coalescing including small aggregates of plasma cells and lymphocytes and macrophages, with and without neutrophils. **E**: skin of the control mice.
(TIF)

S2 Fig Illustration of antibodies derived from the major structural subunit and the minor tip subunit against adherence of a bacteria fimbrial adherence to host cells. (TIF)

S3 Fig Mouse serum antibody adherence inhibition against non-specific *E*. *coli* strain DH5α showed no difference among the control group, the group immunized with dmLT adjuvant alone (without CFA antigen), and the group immunized with major subunit CFA/I/II/IV MEFA adjuvanted with dmLT. Numbers of DH5α bacteria (CFUs; x10^3^) adherent to Caco-2 cells were counted and presented. Data were analyzed using Kruskal-Wallis test, followed by Dunn’s pairwise comparison. The line in each group represents the median with range.
(TIF)
