# Supplementary figures and images for: Antibodies induced by enterotoxigenic Escherichia coli (ETEC) adhesin major structural subunit and minor tip adhesin subunit equivalently inhibit bacteria adherence in vitro
Source: PLoS One. 2019 May 1;14(5):e0216076. doi: 10.1371/journal.pone.0216076 (PMC6493741; doi:10.1371/journal.pone.0216076)

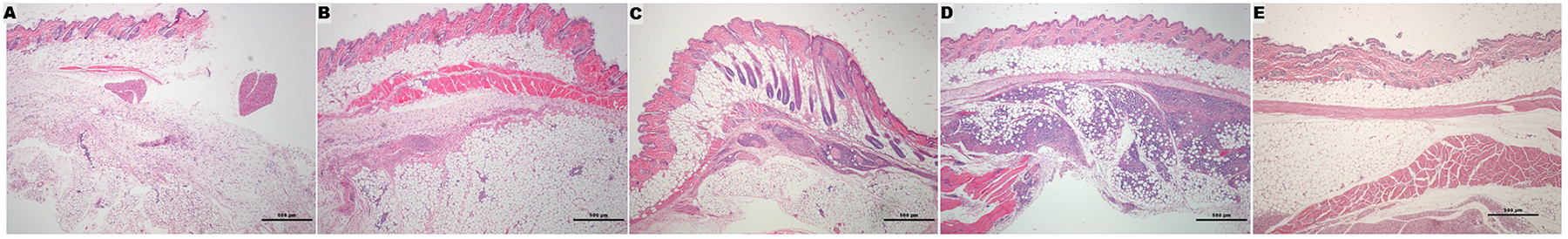

Supplement: S1 Fig — A: skin of a mouse SC immunized with the tip adhesin MEFA (9450) alone. B: skin of mice SC immunized with tip adhesin MEFA (9450) combined with toxoid fusion 3xSTaN12S-mnLTR192G/L211A (9471). Both images are showing not readily apparent localized inflammation. C: skin of a mouse SC immunized with the major subunit CFA/I/II/IV MEFA (9472) alone. D: skin of mice SC immunized with the major subunit CFA/I/II/IV MEFA (9472) combined with toxoid fusion 3xSTaN12S-mnLTR192G/L211A (9471). The inflammation is coalescing including small aggregates of plasma cells and lymphocytes and macrophages, with and without neutrophils. E: skin of the control mice. (TIF) [file pone.0216076.s001.tif]

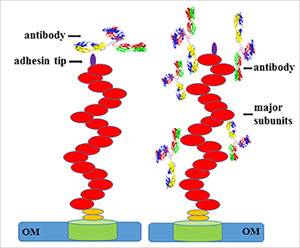

Supplement: S2 Fig — (TIF) [file pone.0216076.s002.tif]

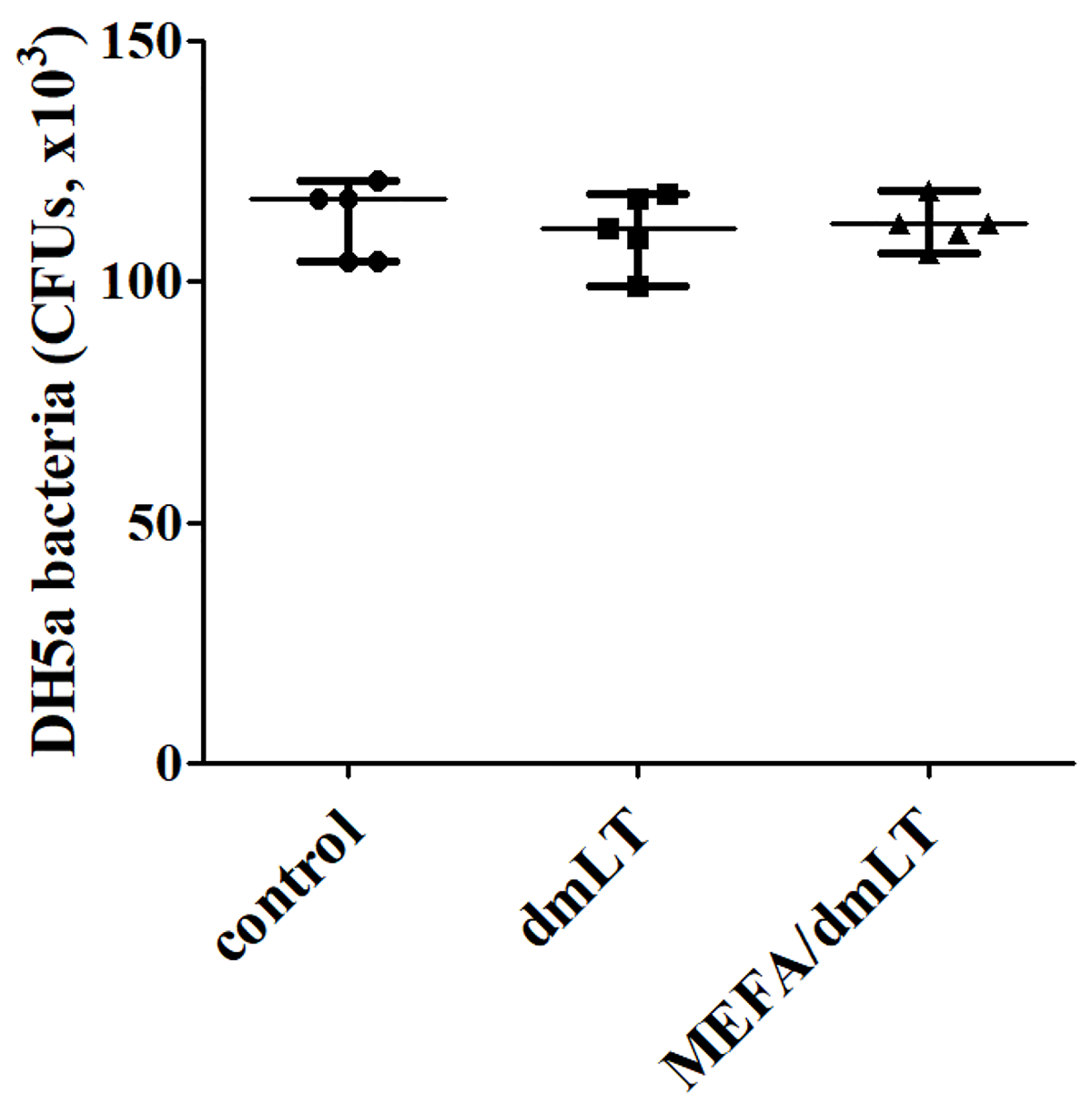

Supplement: S3 Fig — Numbers of DH5α bacteria (CFUs; x103) adherent to Caco-2 cells were counted and presented. Data were analyzed using Kruskal-Wallis test, followed by Dunn’s pairwise comparison. The line in each group represents the median with range. (TIF) [file pone.0216076.s003.tif]
